# Supplementary material for: Non-flipping DNA glycosylase AlkD scans DNA without formation of a stable interrogation complex
Source: Commun Biol. 2021 Jul 15;4:876. doi: 10.1038/s42003-021-02400-x (PMC8282808; doi:10.1038/s42003-021-02400-x)
Supplement: Supplementary file 2 — Description of Additional Supplementary Files [file 42003_2021_2400_MOESM2_ESM.pdf]

**Description of Additional Supplementary Files**

File Name: Supplementary Movie 1
Description: concatenation of exemplary AlkD trajectories while scanning DNA.

File Name: Supplementary Movie 2
Description: concatenation of exemplary AlkF trajectories while scanning DNA.

File Name: Supplementary Movie 3
Description: concatenation of exemplary AlkF-Δpos trajectories while scanning DNA.
