# Supplementary material for: Non-flipping DNA glycosylase AlkD scans DNA without formation of a stable interrogation complex
Source: Commun Biol. 2021 Jul 15;4:876. doi: 10.1038/s42003-021-02400-x (PMC8282808; doi:10.1038/s42003-021-02400-x)
Supplement: Supplementary file 6 — Reporting Summary [file 42003_2021_2400_MOESM6_ESM.pdf]

nature research

Corresponding author(s): Bjørn Dalhus

Last updated by author(s): Jun 15, 2021

Reporting Summary

Nature Research wishes to improve the reproducibility of the work that we publish. This form provides structure for consistency and transparency in reporting. For further information on Nature Research policies, see our Editorial Policies and the Editorial Policy Checklist.

Statistics

For all statistical analyses, confirm that the following items are present in the figure legend, table legend, main text, or Methods section.

n/a	Confirmed	
☒	☐	The exact sample size (*n*) for each experimental group/condition, given as a discrete number and unit of measurement
☒	☐	A statement on whether measurements were taken from distinct samples or whether the same sample was measured repeatedly
☒	☐	The statistical test(s) used AND whether they are one- or two-sided<br>*Only common tests should be described solely by name; describe more complex techniques in the Methods section.*
☒	☐	A description of all covariates tested
☒	☐	A description of any assumptions or corrections, such as tests of normality and adjustment for multiple comparisons
☒	☐	A full description of the statistical parameters including central tendency (e.g. means) or other basic estimates (e.g. regression coefficient) AND variation (e.g. standard deviation) or associated estimates of uncertainty (e.g. confidence intervals)
☒	☐	For null hypothesis testing, the test statistic (e.g. *F*, *t*, *r*) with confidence intervals, effect sizes, degrees of freedom and *P* value noted<br>*Give P values as exact values whenever suitable.*
☒	☐	For Bayesian analysis, information on the choice of priors and Markov chain Monte Carlo settings
☒	☐	For hierarchical and complex designs, identification of the appropriate level for tests and full reporting of outcomes
☒	☐	Estimates of effect sizes (e.g. Cohen's *d*, Pearson's *r*), indicating how they were calculated

Our web collection on statistics for biologists contains articles on many of the points above.

Software and code

Policy information about availability of computer code

Data collection	Micro-manager (version 1.4.22)

Data analysis	FIJI (version 1.51)<br><br>ThunderSTORM plugin<br>(Ovesný, M., Křížek, P., Borkovec, J., Švindrych, Z. & Hagen, G. M. ThunderSTORM: A comprehensive ImageJ plug-in for PALM and STORM data analysis and super-resolution imaging. Bioinformatics 30, 2389–2390 (2014) & Schindelin, J. et al. Fiji: an open-source platform for biological-image analysis. Nat. Methods 9, 609 676–682 (2012))<br><br>TrackMate plugin<br>(Tinevez, J. Y. et al. TrackMate: an open and extensible platform for single-particle tracking. Methods 115, 80–90 (2017))<br><br>R (version 4.0.2)<br><br>A custom-written code in R based upon previous reports<br>(Ahmadi, A., Dalhus, B. & Rowe, A. D. Analysis of DNA scanning by EndoV and hOGG1 proteins: The source code. (2018). doi:https://doi.org/10.5281/zenodo.1487773)<br>(Ahmadi, A. et al. Breaking the speed limit with multimode fast scanning of DNA by Endonuclease V. Nat. Commun. 9, 5381 (2018))<br><br>vbSPT (Persson, F., Lindén, M., Unoson, C. & Elf, J. Extracting intracellular diffusive states and transition rates from single-molecule tracking data. Nat. Methods 10, 265–9 (2013))

For manuscripts utilizing custom algorithms or software that are central to the research but not yet described in published literature, software must be made available to editors and reviewers. We strongly encourage code deposition in a community repository (e.g. GitHub). See the Nature Research guidelines for submitting code & software for further information.

Data

Policy information about availability of data

All manuscripts must include a data availability statement. This statement should provide the following information, where applicable:

- Accession codes, unique identifiers, or web links for publicly available datasets
- A list of figures that have associated raw data
- A description of any restrictions on data availability

The data in the form of sequences of images captured for this study are available from the corresponding authors upon request.

Field-specific reporting

Please select the one below that is the best fit for your research. If you are not sure, read the appropriate sections before making your selection.

☒ Life sciences ☐ Behavioural & social sciences ☐ Ecological, evolutionary & environmental sciences

For a reference copy of the document with all sections, see nature.com/documents/nr-reporting-summary-flat.pdf

Life sciences study design

All studies must disclose on these points even when the disclosure is negative.

Sample size	No sample-size calculations were performed
Data exclusions	As described in the method, trajectories of proteins were detected among sequences of image that were recorded continuously. The frames of datasets with no trajectory detected were excluded from the rest of analysis.
Replication	The interaction of proteins with DNA was recorded for several samples of DNA and proteins and analysis of this interaction was performed on all these data combined.
Randomization	Randomization of samples is not relevant and not performed in this study
Blinding	Blinding is not relevant and not performed in this study

Reporting for specific materials, systems and methods

We require information from authors about some types of materials, experimental systems and methods used in many studies. Here, indicate whether each material, system or method listed is relevant to your study. If you are not sure if a list item applies to your research, read the appropriate section before selecting a response.

Materials & experimental systems

n/a	Involved in the study
☐	☒ Antibodies
☒	☐ Eukaryotic cell lines
☒	☐ Palaeontology and archaeology
☒	☐ Animals and other organisms
☒	☐ Human research participants
☒	☐ Clinical data
☒	☐ Dual use research of concern

Methods

n/a	Involved in the study
☒	☐ ChIP-seq
☒	☐ Flow cytometry
☒	☐ MRI-based neuroimaging

Antibodies

Antibodies used	Anti-digoxygenine (11214667001, Fab fragments from sheep, Roche)<br>Streptavidine (85878-5MG, Sigma-Aldrich)
Validation	The polyclonal antibodies from sheep show 100% reactivity with digoxigenin and digoxin, but no cross-reactivity with other steroids, such as human estrogens (e.g., estradiol) or androgens (e.g., testosterone).
